# Maternal mental health and well-being during the COVID-19 pandemic in Beijing, China

**DOI:** 10.1007/s12519-021-00439-8

**Published:** 2021-06-25

**Authors:** Zhuang Wei, Ming-Yue Gao, Mary Fewtrell, Jonathan Wells, Jin-Yue Yu

**Affiliations:** 1 Department of Child Healthcare, Beijing Children’s HospitalCapital Medical UniversityNational Center for Children’s Health, Beijing, China; 2 Population, Policy and Practice Research and Teaching Department, UCL Great Ormond Street Institute of Child Health, 30 Guilford Street, London, WC1N 1EH UK

**Keywords:** Coping, Coronavirus disease 2019, Maternal health, Mental health, Postpartum

## Abstract

**Background:**

The aim of this study is to evaluate the impact of the coronavirus disease 2019 (COVID-19) pandemic on breastfeeding women and to identify predictors of maternal mental health and coping.

**Methods:**

Mothers aged ≥ 18 years with a breast-fed infant ≤ 18 months of age during the COVID-19 pandemic in Beijing, China, completed a questionnaire. Descriptive analysis of lockdown consequences was performed and predictors of these outcomes were examined using stepwise linear regression.

**Results:**

Of 2233 participants, 29.9%, 20.0% and 34.7% felt down, lonely, and worried, respectively, during the lockdown; however, 85.3% felt able to cope. Poorer maternal mental health was predicted by maternal (younger age, higher education) and infant (older age, lower gestation) characteristics, and social circumstances (husband unemployed or working from home, receiving advice from family, having enough space for the baby, living close to a park or green space). Conversely, better maternal mental health was predicted by higher income, employment requiring higher qualifications, more personal space at home, shopping or walking > once/week and lack of impact of COVID-19 on job or income. Mothers with higher education, more bedrooms, fair division of household chores and attending an online mother and baby group > once/week reported better coping.

**Conclusion:**

The findings highlight maternal characteristics and circumstances that predict poorer mental health and reduced coping which could be used to target interventions in any future public health emergencies requiring social restrictions.

**Supplementary Information:**

The online version contains supplementary material available at 10.1007/s12519-021-00439-8.

## Introduction

From December 2019, a new coronavirus pneumonia (coronavirus disease 2019, COVID-19), caused by severe acute respiratory syndrome coronavirus 2 (SARS-CoV-2) spread globally [1]. To control the pandemic, most countries implemented control measures, including restrictions on mass gathering, quarantine, community control, and business and school closures [2–4]. In 2020, the Chinese Government extended the Lunar New Year holiday from 31st January to 10th March in Hubei and to 9th February in other provinces. People were encouraged to stay at home unless going outside for essential shopping [3]. The lockdown measures effectively delayed virus spread, and prevention measures were adjusted to a lower level from August in Beijing [5]. However, the measures led to considerable disruption to daily life, and many individuals faced unprecedented psychological distress [6].

Pregnant and postpartum women have been reported to be a vulnerable group and they are among those who are most worried about getting infected with COVID-19 [7, 8]. Whilst not apparently at greater risk of infection, they may be more vulnerable to psychological and practical impacts of public health measures that interfere with their practical and psychosocial needs. Studies found that lockdown measures during the COVID-19 led to a reduction in support from relatives and friends, increasing financial difficulties, and increasing risk of domestic violence [7–9]. Moreover, mental health problems during pregnancy may be associated with adverse maternal and infant outcomes including suicidal ideation and impaired mother–infant bonding [10].

In Beijing, routine post-natal home visits were replaced by telephone consultations. This effectively protected pregnant and postpartum women from infection, but the effects of these policies on their psychological status and wellbeing are unknown [11]. Since stress and anxiety in the postpartum period can negatively impact both mother and infant [12], it is important to understand the psychological repercussions of this pandemic and lockdown on maternal health. Results from the UK COVID-19 New Mum Study [13] showed that a high proportion of new mothers reported low mood, anxiety, and loneliness. A survey in Turkey reported the prevalence of postpartum depression was 34% during the pandemic [14]. In China, a national survey in the general population indicated that depression, anxiety, insomnia, and acute stress might have been common during the pandemic [6].

Based on the experiences from previous flu pandemics, ongoing long-term monitoring of pregnant and postpartum women is needed following the COVID-19 pandemic [15]. However, there is little research on the psychological status of these vulnerable populations during the pandemic, especially breastfeeding women [16]. Compared to formula-feeding mothers, breastfeeding outcomes may be more dependent on family and health professional advice and support. Moreover, successful breastfeeding is of great importance for maternal and infant health, both short and longer term. Hence, this study aimed to investigate the psychological status and well-being of mothers who were breastfeeding during the COVID-19 lockdown period in Beijing using an adapted version of the UK COVID-19 New Mum Survey. This information is important to identify issues experienced by this vulnerable group that may require intervention, and may also contribute to the formulation of public health policies in the event of future public health emergencies.

## Methods

The study was conducted from August 1st to October 31st, 2020, at Shunyi Maternal and Children’s Hospital of Beijing Children’s Hospital, China. Advertisements, including a brief introduction and inclusion criteria, were posted on the bulletin board in the clinic reception. Eligible mothers were invited to complete a hard copy of the one-time, anonymous questionnaire. The questionnaire, containing 48 questions, was completed at the clinic and took approximately 15–20 minutes. Maternal inclusion criteria were (1) age ≥ 18 years; (2) infant < 18 months at the time of survey completion; (3) living in Beijing and breastfeeding their infant (exclusively or partially) for some or all of the lockdown period (January 23rd to July 31st, 2020). An online version of the questionnaire was also shared via relevant professional groups and contacts, and via word of mouth. Mothers who were eligible and interested could start the survey by clicking the link https://wenjuan.net/s/NVveyew/ or scanning a QR code.

The questionnaire was adapted from the UK COVID-19 New Mum Survey [13, 17], translated by a Chinese team member and refined by a pediatrician with clinical experience of infant feeding. A repeated forward–backward translation procedure was adopted. We first forward translated the original English version to a Chinese version, and then the Chinese version was translated back into English and compared to the original English version. Errors in the Chinese version were identified through changes in meaning that arose in the back translation. Women were asked to answer the questions for the period of lockdown. The survey (see Supplementary Questionnaire) had four parts: (1) demographic characteristics; (2) infant feeding and birth experiences; (3) maternal mood, activities, living circumstances and access to support during the lockdown; (4) impact of COVID-19 on maternal perceptions of their mental health, wellbeing and life patterns during the lockdown period. Due to ethical considerations in this anonymous survey where there was no further contact with participants, formal depression and anxiety assessment was not performed. Instead, we included questions on recent emotions and the influence of lockdown on daily life and work.

Ethical approval was obtained from the Beijing Children’s Hospital Research Ethics committee (2020-Z-102). Informed consent to participate in the study was obtained from participants. Mothers started the survey by choosing “yes” for the first question, which asked for consent; they could refuse to answer any question if they did not want to.

Survey data were manually entered by independent research assistants, and checked by the principal researcher. Online data were exported from https://wenjuan.net/s/NVveyew/. Statistical analysis was conducted in SPSS version 25.0 (IBM., Armonk, NY, USA). Descriptive analysis was performed to report mean ± standard deviation or number (percentage) of demographic characteristics, maternal perceptions, support received, activities and consequences of lockdown. Principal components analysis (PCA) was performed for maternal perceptions and was conducted on the correlation matrix of the survey responses (Supplementary Table 1); PCA performance was assessed through Kaiser–Meyer–Olkin measure of sampling adequacy (0.913) and Bartlett test of sphericity (*P* < 0.05). Eigenvalues (of 1), scree plots, and parallel analysis were used to identify the components of perceptions (Supplementary Fig. 1).

**Fig. 1:**
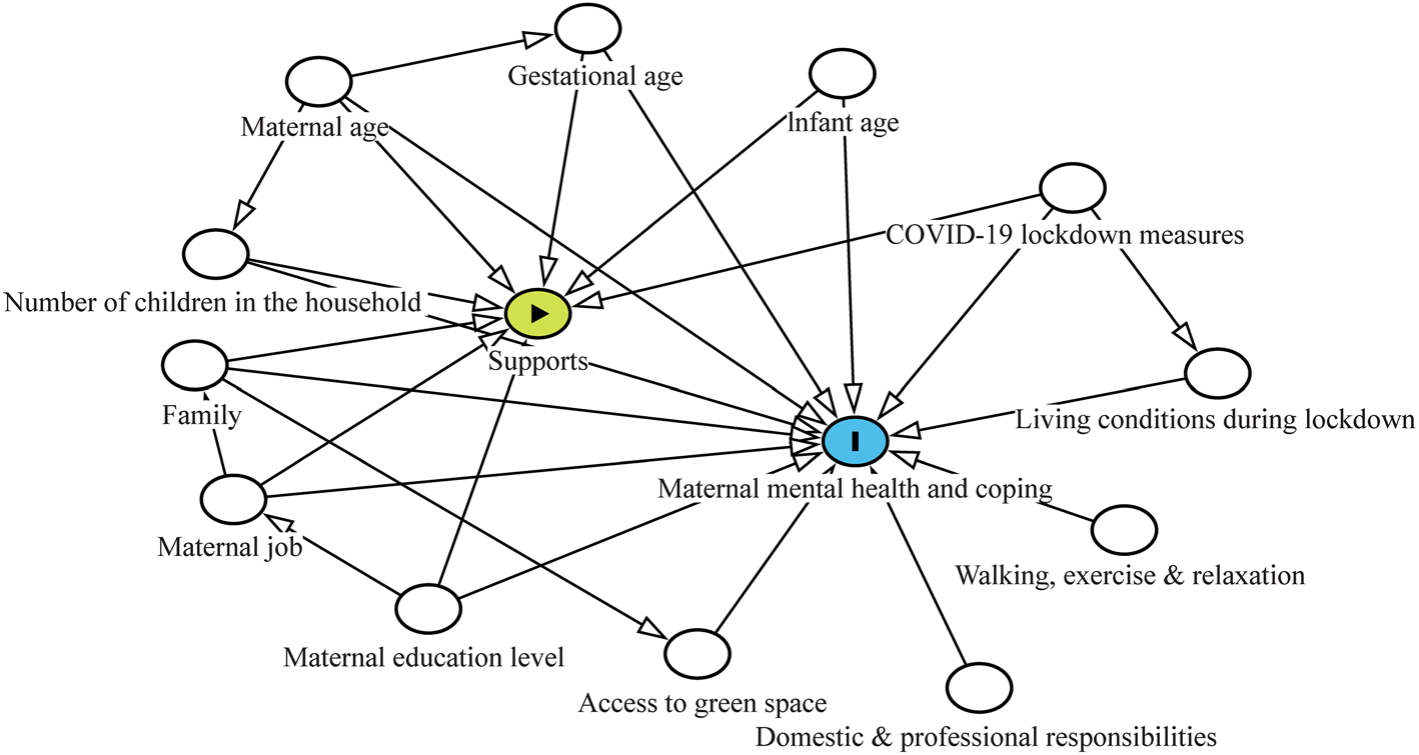
Relationship between maternal mental health or coping and activities, support and consequences of lockdown. The model was generated using the DAGitty version 3.0 (online at https://dagitty.net/dags.html)

Stepwise linear regression was applied to investigate predictors of maternal mental health and coping (outcomes), including infant and maternal socio-economic status, support, activities and impact of COVID-19, which was identified using the directed acyclic graphs (DAGitty version 3.0, Fig. 1). 95% confidence intervals (CI) were presented for regression coefficients. *P* < 0.05 was considered statistically significant.

## Results

From 1st August to 31st October 2020, 2233 participants returned the questionnaire, including 2179 hard copies and 54 online submissions. There were 1241 and 992 mothers who gave birth to a child before and during the lockdown period, respectively. Mean participant age was 31.4 ± 4.1 years, and mean infant age 8.2 ± 4.0 months at the time of survey completion (range 1–18 months). All participants were married, with only 17 living alone during the lockdown period. Background characteristics are shown in Table 1.

**Table 1:** Background characteristics of the study population (*N* = 2233)

Characteristics	Valid *n*	Values
Maternal age (y), mean ± SD	2193	31.4 ± 4.1
Infant age (mon), mean ± SD	2222	8.2 ± 4.0
Infant gestation (wk), mean ± SD	2199	38.8 ± 1.5
Infant gender, *n* (%)	2229	
Male		1128 (50.6)
Female		1101 (49.4)
Delivery method, *n* (%)	2227	
Vaginal		1462 (65.6)
Caesarean		765 (34.4)
Have skin to skin contact after birth, *n* (%)		2154 (96.6)
Primiparous mother, *n* (%)		1420 (64.1)
Total years of full-time education, mean ± SD		15.44 ± 2.60
Maternal education, *n* (%)	2205	
Diploma or under		927 (42.0)
Bachelor’s degree		929 (42.1)
Master’s degree		318 (14.4)
PhD/professional qualification		31 (1.4)
Living conditions^a^, *n* (%)	2038	
Crowded apartment with community garden		477 (23.4)
Crowded apartment without community garden		286 (14.0)
Spacious apartment with community garden		939 (46.1)
Spacious apartment without community garden		277 (13.6)
House		19 (0.9)
Villa		40 (2.0)
Living status during the lockdown, *n* (%)	2191	
With husband		800 (36.5)
With husband and parents		562 (25.7)
With husband and parents-in-law		805 (36.7)
Alone		17 (0.8)
Other		7 (0.3)
Household income in CNY, *n (%)*	2213	
< 200,000		738 (33.3)
< 300,000		539 (24.4)
< 400,000		291 (13.1)
< 500,000		104 (4.7)
> 500,000		67 (3.0)
Other		22 (1.0)
Prefer not to say		452 (20.4)

*SD* standard deviation. ^a^Spacious apartment having less than 3 resident units on each floor; crowded apartment as reference (normally have 8–20 resident unit on each floor)

Four PCA components were identified from maternal perceptions (Table 2): (1) “maternal mental health”, including physical symptoms (tiredness, low appetite, and bad sleep) and perceptions of mood (loneliness, relaxation, annoyance, and worrying), as shown in Supplementary Table 1. A higher score indicates poorer mental health while a lower score indicates better mental health; (2) “coping”, including chatting with others, enjoying the changing seasons, being able to focus on your health, and perception of coping well; (3) “positive reaction to surroundings”, including feeling close to the community and increased appetite; (4) “time to focus on interests”, including exercise and hobbies during the lockdown. In the present study, the focus was on the mental health and coping components as outcomes.

**Table 2:** Descriptive results of each item from the principal component analysis (*N* = 2233)

Psychological status and daily activities of participants during the lockdown	Valid *n*	Not at all	To a small extent	To some extent	To a high extent
Negative body reactions^a^					
I've been feeling tired or having little energy	2211	1465 (66.3)	547 (24.7)	159 (7.2)	40 (1.8)
I’ve had trouble falling or staying asleep	2210	1488 (67.3)	504 (22.8)	168 (7.6)	50 (2.3)
I’ve been having a poor appetite	2215	1554 (70.2)	450 (20.3)	184 (8.3)	27 (1.2)
I’ve been feeling down	2209	1548 (70.1)	474 (21.5)	149 (6.7)	38 (1.7)
I’ve been feeling lonely	2209	1766 (79.9)	299 (13.5)	115 (5.2)	29 (1.3)
I’ve had trouble relaxing	2211	1445 (65.4)	573 (25.9)	158 (7.1)	35 (1.6)
I’ve become easily annoyed or irritable	2214	1388 (62.7)	611 (27.6)	173 (7.8)	42 (1.9)
I’ve been feeling worried	2207	1439 (65.3)	561 (25.4)	164 (7.4)	43 (1.9)
Coping^b^					
I’ve had the opportunity to chat with my family and friends	2208	153 (6.9)	542 (24.5)	957 (43.3)	556 (25.2)
I’ve enjoyed the spring weather	2205	544 (24.7)	813 (36.9)	618 (28.0)	230 (10.4)
I feel able to cope with the situation	2205	324 (14.7)	707 (32.1)	724 (32.8)	450 (20.4)
I’ve had time to focus on my health	2202	381 (17.3)	882 (40.1)	730 (33.2)	209 (9.5)
Positive reaction to surroundings^c^					
I feel connected with my local community	2190	615 (28.1)	919 (42.0)	525 (24.0)	131 (6.0)
I’ve been having an increased appetite	2216	1434 (64.7)	487 (22.0)	245 (11.1)	50 (2.3)
Time to focus on interests^d^					
I’ve had time to enjoy personal interests	2212	841 (38.0)	785 (35.5)	464 (21.0)	122 (5.5)
I’ve had time to exercise	2207	651 (29.5)	915 (41.5)	535 (24.2)	106 (4.8)

Values are given as *n* (%). ^a, b, c, d^These items were engendered from the principal component analysis. The principal component analysis was conducted on the correlation matrix of the survey responses. Item “Feeling that housework chores are fairy assigned” in the original questionnaire (question 47) was not included in the analysis, instead, it was analyzed as a covariance in the regression

More than half of the participants reported no negative physical symptoms or emotions during the lockdown period (Table 2). Less than 10% reported extent feeling tired, difficulty falling asleep, having low mood, being lonely, irritable, worried or having trouble relaxing to more than a small extent. More than half of the mothers felt able to cope with the situation and 68.5% felt they had more opportunity to chat with friends and family to some or a high extent. However, the majority either did not, or to a small extent, feel close to their community (70.1%), have time to enjoy personal interests (73.5%) and have time to exercise (71.0%).

Most mothers (90.6%) did not travel for work; however, 54.5% walked or exercised outside more than 4–5 times per week. Online activities, such as shopping, playing games, etc., were reported by 43.8%, while 39.9% used a relaxation technique; the most common being yoga, listening to relaxing music and meditation.

The majority (91.3%) of mothers received support from relatives during the pandemic and 35.8% reported the household chores were more equally assigned to some or a high extent (Table 3). Fewer than half (48.1%) contacted a baby or breastfeeding group 1–5 times per week, and 9.3% had contact at least daily. Over half the sample (60.3%) of participants had no contact with health professionals.

**Table 3:** Support measures for mothers during lockdown (*N* = 2233)

Variables	Valid *n*	Values
Got or getting enough support and help with own health	2102	
Yes		1920 (91.3)
No		182 (8.7)
Any advice about infant feeding provided by professionals?	2226	
Yes		2145 (96.4)
No		81 (3.6)
Any advice about infant feeding provided by family?	2226	
Yes		2134 (95.9)
No		92 (4.1)
Had contact with a mother and baby or breastfeeding support group (times/wk)	2207	
0 times		940 (42.6)
1–3 times		894 (40.5)
4–5 times		168 (7.6)
Daily or more		205 (9.3)
Had contact with a health professional (times/wk)	2204	
0 times		1330 (60.3)
1–3 times		699 (31.7)
4–5 times		81 (3.7)
Daily or more		94 (4.3)
I feel the household chores are more equally divided among household members	2203	
Not at all		637 (28.9)
Very little		778 (35.3)
To some extent		652 (29.6)
To a high extent		136 (6.2)

Values are given as *n* (%)

In linear regression analysis, poorer maternal mental health was predicted by both maternal (younger age, higher education) and infant (lower gestational age, older age at the time of survey completion) characteristics, as well as by social circumstances (husband unemployed or working from home, receiving advice from family, having enough space for the baby, living close to a park or green space). Conversely, better maternal mental health was predicted by higher income, having a job requiring higher qualifications, having more personal space at home, shopping or walking more than once a week and lack of impact of COVID-19 on job or income (Table 4).

**Table 4:** Multivariate linear regression on predictors of maternal mental health

Variables	*B*	95% CI for *B*		
		Lower	Upper	Sig
Infant and maternal SES				
Infant gender^a^	0.066	− 0.032	0.163	0.186
Maternal age	− **0.018**	− **0.031**	− **0.005**	**0.008**
Infant age	**0.014**	**0.000**	**0.027**	**0.044**
Infant GA	− **0.032**	− **0.064**	**0.000**	**0.051**
Income 20,000–30,000 CNY^b^	− 0.085	− 0.210	0.040	0.182
Income > 30,000 CNY	− **0.303**	− **0.447**	− **0.160**	**0.000**
Bachelor^c^	**0.148**	**0.032**	**0.264**	**0.012**
Master and Doctor	0.158	− 0.010	0.326	0.066
UK job level III^d^	− **0.201**	− **0.350**	− **0.053**	**0.008**
UK job level I and II	− **0.185**	− **0.329**	− **0.042**	**0.011**
One child totally^e^	− 0.091	− 0.201	0.018	0.103
Living conditions				
Spacious apartment^f^	− **0.096**	− **0.200**	**0.007**	**0.068**
Total bedrooms	0.042	− 0.012	0.096	0.124
Enough space for baby^g^	**0.171**	**0.010**	**0.332**	**0.038**
Close to the square/park^h^	**0.420**	**0.273**	**0.567**	**0.000**
Close to the square and park	**0.185**	**0.051**	**0.319**	**0.007**
Not crowded home^i^	− **0.380**	− **0.484**	− **0.275**	**0.000**
COVID-19 impacts^j^				
Income				
Moderate	− 0.161	− 0.365	0.044	0.124
Little	− 0.172	− 0.412	0.068	0.161
No	− **0.447**	− **0.711**	− **0.183**	**0.001**
Job				
Moderate	− **0.264**	− **0.424**	− **0.104**	**0.001**
Little	− **0.322**	− **0.527**	− **0.117**	**0.002**
No	− **0.333**	− **0.506**	− **0.159**	**0.000**
Husband job				
Work at home	**0.239**	**0.091**	**0.388**	**0.002**
Have no job	**0.430**	**0.120**	**0.740**	**0.007**
No impact	0.035	− 0.128	0.198	0.673
Buying essentials				
Moderate	− 0.002	− 0.252	0.247	0.984
Little	− 0.088	− 0.367	0.190	0.535
No	0.097	− 0.232	0.426	0.563
Buying food				
Moderate	− 0.002	− 0.200	0.197	0.988
Little	− 0.041	− 0.280	0.199	0.740
No	− 0.084	− 0.384	0.217	0.586
Buying medicines				
Moderate	− 0.068	− 0.294	0.157	0.552
Little	− 0.046	− 0.294	0.202	0.716
No	− 0.074	− 0.361	0.213	0.614
Supports				
Received professional advice^k^	− 0.260	− 0.563	0.044	0.093
Received family advice^l^	**0.341**	**0.046**	**0.636**	**0.023**
Fair household chores assign^m^	**0.281**	**0.179**	**0.382**	**0.000**
Consulting health > 1/wk^n^	0.034	− 0.082	0.150	0.570
Discuss health in group > 1/wk^o^	− 0.035	− 0.152	0.081	0.551
Activities				
Go shopping > 1/wk^p^	− **0.114**	− **0.222**	− **0.006**	**0.038**
Walking for exercise > 1/wk^q^	− **0.410**	− **0.570**	− **0.249**	**0.000**
Travel for work > 1/wk^r^	**0.475**	**0.292**	**0.658**	**0.000**
Use relaxation technique > 1/wk^s^	− 0.020	− 0.129	0.089	0.714

Component includes low mood, lonely, not relaxed, annoyed, worried, low appetite, bad sleep, tired; negative *B* value means that a higher score of the predictor is associated with better maternal mental health (a lower negative impact on maternal mental health). Significant values were shown in bold font. *CI* confidence interval, *SES* socio-economic status, *GA* gestational age, *COVID-19* coronavirus disease 2019. ^a^Boy as reference; ^b^income < 200,000 CNY as reference; ^c^diploma and under as reference; ^d^UK job class IV and V as reference. Class IV and V mainly refers to manual labor jobs which do not require higher education; jobs in class III and II require certain training, certifications, licenses and degrees to qualify (e.g., editor, police officer, nurse); jobs in class I involve professional careers which require advanced degrees, high-end skills or expertise (e.g., engineer, physician, lawyer); ^e^more than one child as reference; ^f^spacious apartments have less than 3 resident units on each floor, crowded apartments as reference (normally 8–20 resident units on each floor); ^g^not enough space as reference; ^h^not close to square or park as reference; ^i^crowded home as reference; ^j^major impact on the following items (income, job, husband’s job, buying essentials, buying food, buying medicines) as reference; ^k^no professional advice received as reference; ^l^no family advice received as reference; ^m^household chores assigned not fair as reference; ^n, o, p, q, r, s^no impact as reference

Mothers with a bachelor degree, more bedrooms at home, fair division of household chores and attending an online mother and baby group more than once a week reported better coping. In contrast, living within walking distance of a square or park, consulting for a health issue, using relaxation therapy or going shopping more than once a week was associated with worse coping (Table 5).

**Table 5:** Multivariate linear regression on predictors of coping

Variables	*B*	95% CI for *B*		
		Lower	Upper	Sig
Infant and maternal SES				
Infant gender^a^	0.006	− 0.096	0.108	0.911
Maternal age	0.004	− 0.009	0.018	0.545
Infant age	− 0.013	− 0.027	0.001	0.072
Infant GA	0.018	− 0.015	0.052	0.284
Income 20,000–30,000 CNY^b^	0.009	− 0.121	0.140	0.889
Income > 30,000 CNY	− 0.080	− 0.229	0.069	0.293
Bachelor^c^	**0.213**	**0.092**	**0.334**	**0.001**
Master and Doctor	0.143	− 0.033	0.318	0.111
UK job level III^d^	− 0.017	− 0.172	0.138	0.831
UK job level I and II	− 0.084	− 0.234	0.066	0.271
One child totally^e^	− 0.064	− 0.178	0.051	0.276
Living conditions				
Spacious apartment^f^	− 0.006	− 0.114	0.102	0.917
Total bedrooms	**0.063**	**0.007**	**0.119**	**0.027**
Enough space for baby^g^	0.046	− 0.122	0.214	0.589
Close to the square/park^h^	− **0.279**	− **0.432**	− **0.126**	**0.000**
Close to the square and park	− 0.092	− 0.232	0.048	0.199
Not crowded home^i^	− **0.132**	− **0.241**	− **0.023**	**0.018**
COVID-19 impacts^j^				
Income				
Moderate	− 0.088	− 0.302	0.126	0.419
Little	− 0.070	− 0.321	0.181	0.584
No	7.818E−05	− 0.276	0.276	1.000
Job				
Moderate	0.079	− 0.088	0.246	0.356
Little	− 0.064	− 0.278	0.149	0.555
No	− 0.011	− 0.192	0.170	0.908
Husband job				
Work at home	0.069	− 0.086	0.225	0.382
Have no job	0.242	− 0.082	0.565	0.144
No impact	0.084	− 0.086	0.254	0.334
Buying essentials				
Moderate	0.047	− 0.213	0.307	0.722
Little	0.117	− 0.174	0.407	0.431
No	0.239	− 0.104	0.582	0.172
Buying foods				
Moderate	0.135	− 0.072	0.342	0.202
Little	0.044	− 0.206	0.294	0.731
No	0.167	− 0.146	0.481	0.295
Buying medicine				
Moderate	− 0.064	− 0.299	0.172	0.596
Little	0.027	− 0.232	0.286	0.836
No	0.129	− 0.171	0.428	0.399
Supports				
Received professional advice^k^	− 0.024	− 0.341	0.293	0.883
Received family advice^l^	0.145	− 0.162	0.453	0.354
Fair household chores assign^m^	**0.580**	**0.474**	**0.686**	**0.000**
Consulting health > 1/wk^n^	− **0.130**	− **0.251**	− **0.008**	**0.036**
Discuss health in group > 1/wk^o^	**0.146**	**0.024**	**0.268**	**0.019**
Activities				
Go shopping > 1/wk^p^	− **0.131**	− **0.244**	− **0.019**	**0.022**
Walking for exercise > 1/wk^q^	0.131	− 0.036	0.299	0.124
Travel for work > 1/wk^r^	− 0.106	− 0.297	0.084	0.274
Use relaxation technique > 1/wk^s^	− **0.199**	− **0.312**	− **0.085**	**0.001**

Component includes having opportunity to chat with family/friends, enjoying the spring, be able to cope, and concern about own health; negative *B* value means that a higher score of the predictor is associated with worse coping

Significant values were shown in bold font

*CI* confidence interval, *SES* socio-economic status, *GA* gestational age, *COVID-19* coronavirus disease 2019

^a^Boy as reference; ^b^income < 200,000 CNY as reference; ^c^diploma and under as reference; ^d^UK job class IV and V as reference. Class IV and V mainly refers to manual labor jobs which do not require higher education; jobs in class III and II require certain training, certifications, licenses and degrees to qualify (e.g., editor, police officer, nurse); jobs in class I involve professional careers which require advanced degrees, high-end skills or expertise (e.g., engineer, physician, lawyer); ^e^more than one child as reference; ^f^spacious apartments have less than 3 resident units on each floor, crowded apartments as reference (normally 8–20 resident units on each floor); ^g^no enough space as reference; ^h^not close to square or park as reference; ^i^crowded home as reference; ^j^major impact on the following items (income, job, husband’s job, buying essentials, buying food, buying medicines) as reference; ^k^no professional advice received as reference; ^l^no family advice received as reference; ^m^household chores assigned not fair as reference; ^n, o, p, q, r, s^no impact as reference

## Discussion

Maternal mental health problems are increasingly recognized to have potential long-term consequences for mothers and infants. Nearly 20% of mothers experience an episode of depression within the first 3 months postpartum [18], and excessive stress, low sleep quality, and lack of social support are recognized risk factors [19]. Moreover, studies show that maternal psychological status during breastfeeding may influence infant growth and behavior by altering the breastmilk composition and volume [20, 21]. Therefore, maternal mental health during this period, especially during a period of social restriction resulting from a public health emergency, deserves attention. Overall, our findings suggest that the pandemic and resulting lockdown had a limited negative impact on the mental health and well-being of women who were breastfeeding during the 2020 lockdown in Beijing. However, our measure of maternal mental health, which included both physical and psychological components, was predicted by factors related to the mother, infant, living circumstances and the impact of the pandemic.

Higher maternal age, higher income, a job requiring higher qualifications, and living in a spacious apartment without overcrowding were positively associated with mental health, as was younger infant age, and higher gestational age. Surprisingly, higher maternal educational qualifications, having enough space for their baby, and living close to a green space or park were negative predictors of mental health. The explanation for these findings is unclear. It is possible that mothers with higher qualifications were more likely to follow and worry about the latest COVID-19 data. Having enough space for their baby at home could also mean that the mother had less personal space to relax. As for the relationship between green space and mental health, although several studies reported that green spaces may reduce stress [22–24], a longitudinal study showed that they were associated with better mental health among men, but not women [25], presumably because the frequency of using green spaces may differ between genders. Moreover, in the present study, mothers living close to a green space may have been accustomed to using it regularly under normal circumstances, and therefore found it stressful not to be able to do this safely during the pandemic.

Factors related to the impact of the pandemic on living circumstances were also influential; a lack of impact on income or employment predicted better outcomes, as did going shopping or for a walk at least once per week. Conversely, mothers whose partner was working at home or unemployed, who had to travel to work, who received advice from their family, or who perceived that household chores were more equally shared reported worse outcomes. Previous studies showed that a husband’s employment can affect the emotional health of their wife, especially for unemployed men [26]. Interestingly, women who received advice from family reported worse mental health, possibly because perceived interference by family members caused anxiety [27]. Further, although our findings on household chores contrast with previous publications [28, 29], a recent study reported that a spouse’s involvement in housework can engender work–family conflict for both women and men [30]. Interestingly, feeling that chores had become more equally divided during the lockdown predicted poorer mental health but better coping. Possibly the sharing of housework provided practical support, allowing mothers to cope, whilst simultaneously presenting stress due to conflict with the partner.

Our results suggest that coping was associated more with the mother’s living circumstances and activities during the pandemic, than with background characteristics. Thus, mothers who reported having more bedrooms, fair division of household chores and who participated in an online group to discuss health and infant care at least weekly were better able to cope. Conversely, mothers who reported their home was not crowded, who lived close to a green space or park, or who went shopping more than once per week were less able to cope, as were those who consulted a health professional or practiced a relaxation technique more than once per week. It seems likely that some of these associations reflect maternal responses to a perceived inability to cope, rather than a causal effect. For example, mothers who were feeling unable to cope may have been more likely to seek health professional advice or use relaxation therapies. We cannot address the direction of effect in this cross-sectional study. Shopping more than once a week was related to better mental health but worse coping. It is possible that shopping, and the associated outdoor exercise, has positive effects on mental health [31], whilst the need to do so more than once a week could reflect a lack of household support with this chore [32]. Moreover, visiting crowded areas whilst shopping during the pandemic could induce anxiety.

Although longitudinal studies are needed to confirm the direction of effects of lockdown measures and maternal mental health, our results suggested that mothers who have a higher income, employment requiring higher qualifications, more personal space at home, shopping or walking more than once a week and lack of impact of COVID-19 on job or income were less likely to present negative emotion during the lockdown. These reflect that a stable socio-economic status can help to release maternal stress during such public health emergencies. Hence, for lower income mothers, virtual parent and caregiver support groups and helplines are needed, including those targeting single-headed households, to ensure that mental health and psychosocial support during the pandemic. Moreover, there is an urgent need for researchers to develop strategies to mitigate against the impact of lockdown measures for mothers giving birth during the current pandemic or any similar situations in future. Our results might help to focus strategies or interventions on certain groups of mothers who seem to be vulnerable (younger mothers, mothers who have higher education, and mothers who have an infant with older age and/or lower gestation).

Compared with British mothers [13], fewer mothers reported high levels of low mood, anxiety, and loneliness. This may reflect cultural differences in mothers’ resilience to stress and coping, as well as differences in their willingness to report feelings and perceptions [33]. Moreover, unlike the UK study where mothers completed the questionnaire during the lockdown period, the present study was conducted when baby clinics had reopened. Mothers were asked to recall their feelings and experiences during the lockdown period and this may have introduced recall bias. Despite these differences, the present study found that mothers of infants with higher gestational age and those with higher family income had better maternal mental health, consistent with the UK study. Additionally, travelling to work more than once a week predicted worse mental health in both studies. Of 208 healthcare workers in our study, 81% reported moderate to significant feelings of tiredness and low-quality sleep. Similar results were reported from recent surveys in China and Italy, where 50.3% and 44.6% of healthcare workers reported depression and anxiety, respectively [34, 35]. Collectively, these results suggest that healthcare workers, those with lower income or delivering at a lower gestational age may represent higher risk groups for poor mental health during the pandemic.

In addition to potential recall bias, the study has some other limitations. All participants lived in Beijing and might not be representative of new mothers in China; although the population of Beijing includes people from all over the country, measures taken to address the pandemic differed between provinces and cities. Also, due to the anonymous nature of the survey, we did not formally assess depression and anxiety. Nevertheless, a larger proportion of the population does not fit a clinical diagnosis of depression or anxiety but is somewhat at risk, which makes the present results more generalizable. Last but not least, no formal adjustment was made for multiple tests in this analysis, which should be considered when interpreting the results of this study.

In conclusion, our findings highlight maternal and infant characteristics and circumstances associated with poor mental health and reduced coping during the pandemic. These findings could be used to identify vulnerable women who might be the target of interventions and circumstances which could be addressed in any future public health emergencies requiring social restrictions.

## Supplementary Information

Below is the link to the electronic supplementary material.

**Supplementary Fig. 1** Correlation between the principal component and value of the measurements. In the original questionnaire, answers to the mood related questions (to what extend did you experienced the following emotion/symptoms) were coded as “totally not” = 1, “a little” = 2, “moderate” = 3, “large” = 4. **a** Component 1 by sum of each item (in component 1) by same classification direction; **b** Component 2 by sum of each item (in component 2) by same classification direction; **c** Component 3 by sum of each item (in component 3) by same classification direction; **d** Component 4 by sum of each item (in component 4) by same classification direction (TIF 3532 KB)

Supplementary questionnaire (DOCX 26 KB)

Supplementary Table 1 (DOCX 20 KB)
