# Supplementary figures and images for: Maternal mental health and well-being during the COVID-19 pandemic in Beijing, China
Source: World J Pediatr. 2021 Jun 25;17(3):280–9. doi: 10.1007/s12519-021-00439-8 (PMC8231088; doi:10.1007/s12519-021-00439-8)

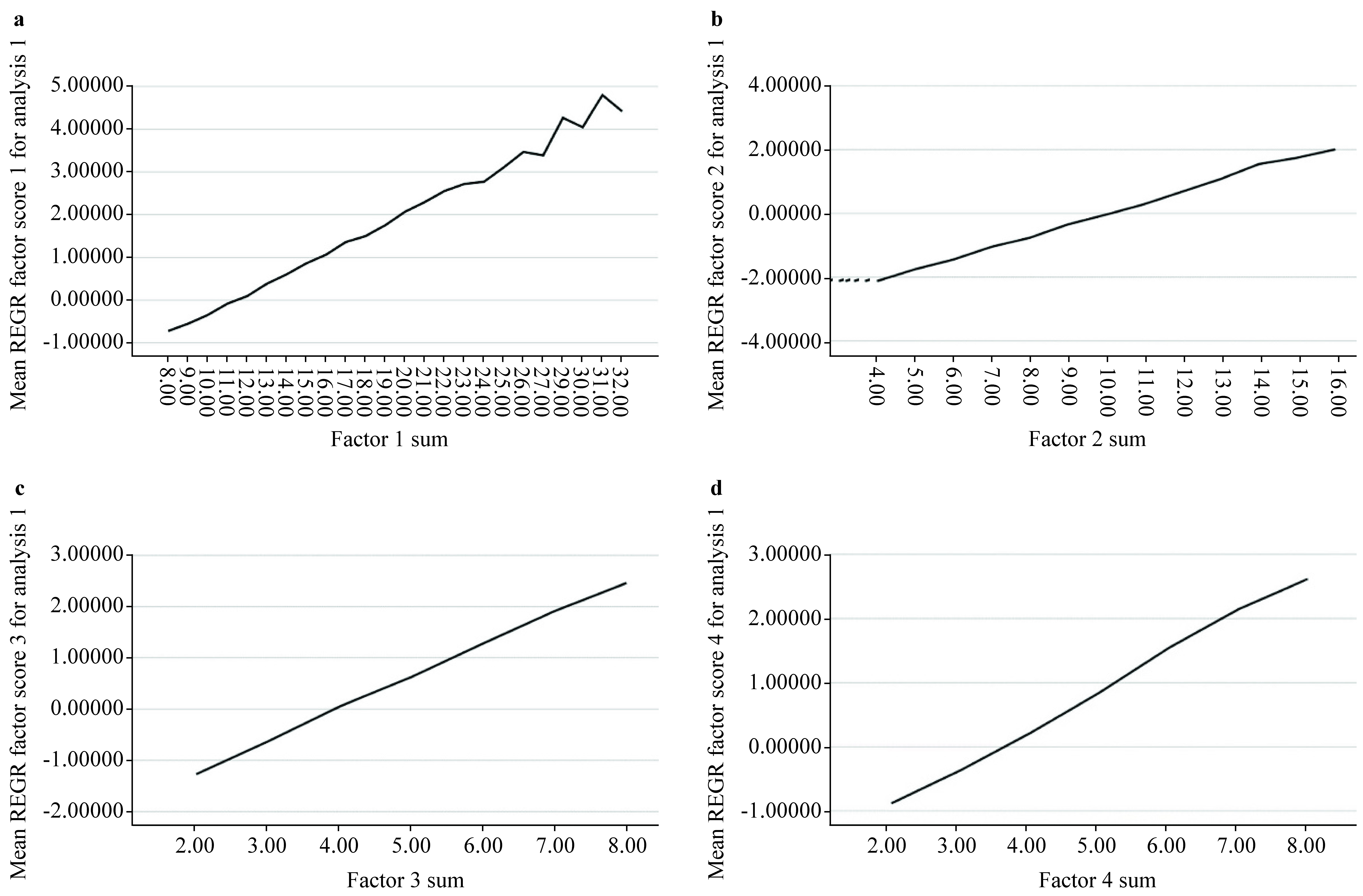

Supplement: Supplementary file 1 — Supplementary Fig. 1 Correlation between the principal component and value of the measurements. In the original questionnaire, answers to the mood related questions (to what extend did you experienced the following emotion/symptoms) were coded as “totally not” = 1, “a little” = 2, “moderate” = 3, “large” = 4. a Component 1 by sum of each item (in component 1) by same classification direction; b Component 2 by sum of each item (in component 2) by same classification direction; c Component 3 by sum of each item (in component 3) by same classification direction; d Component 4 by sum of each item (in component 4) by same classification direction (TIF 3532 KB) [file 12519_2021_439_MOESM1_ESM.tif]
