# Supplementary material for: Maternal mental health and well-being during the COVID-19 pandemic in Beijing, China
Source: World J Pediatr. 2021 Jun 25;17(3):280–9. doi: 10.1007/s12519-021-00439-8 (PMC8231088; doi:10.1007/s12519-021-00439-8)
Supplement: Supplementary file 2 — Supplementary questionnaire (DOCX 26 KB) [file 12519_2021_439_MOESM2_ESM.docx]

Maternal Mental Health and Infant Feeding Survey

(Translated version, originally in Chinese)

Thank you for taking a few minutes to participate in this survey. Please read the following informed consent form carefully, and check the relevant information after reading

The purpose of this questionnaire is to investigate the current feeding situation and family nurturing situation of mothers who are feeding babies under 18 months old, so as to promote breastfeeding and improve the nurturing environment, and provide guidance on feeding. This questionnaire will ask questions about basic conditions, family conditions, baby feeding conditions, and changes in living conditions during the COVID-19 pandemic. The questionnaire does not involve personal information such as names and phone numbers. We will treat the contents of the questionnaire confidentially.

I have fully understood the above information and voluntarily participated in the questionnaire survey

○ Agree

○ Disagree

1. Baby's date of birth (fill in the blanks)

________________________

2. What is the baby's gender? (Single-choice question)

1 Male

2 Female

3. Your age (fill in the blanks)

________________________

4. What is your marital status

1 Married

2 Co-habititing with partner

3 Single

5. The age in months of the baby you are currently feeding is _______ months (Fill in the blank question)

________________________

6. Please fill in the following content (Fill-in-the-blank questions)

How many kilograms was the baby's weight at the last measurement? ________________________

How many months was the baby at that time? ________________________

7. How many weeks of gestation was the baby? (Fill in the blank question)

________________________

8. The delivery method of the baby you are currently feeding is (Single-choice question)

1 Natural childbirth (vaginal)

2 Natural childbirth, forceps assisted delivery

3 Planned caesarean section

4 Emergency caesarean section

9. Your place of delivery was (Single-choice question)

1 Public hospital

2 Private hospital

3 Home

4 Other ____________

10. After you give birth, did you have skin-to-skin contact with your infant? (Single-choice question)

1 Yes

2 No

11. When you give birth, what was your initial planned feeding method? (Single-choice question)

1 Exclusive breastfeeding for up to 6 months

2 Formula feeding

3 Mixed feeding

4 Did not consider much at the time

12. What was your actual feeding method up to 6 months? (Single-choice question)

1 Exclusive breastfeeding

2 Formula feeding

3 Mixed feeding

13. The baby's current feeding method is (Single-choice question)

1 Exclusive breastfeeding

2 Breastfeeding plus complementary food

3 Formula feeding

4 Formula feeding plus complementary food

5 Mixed feeding (exclusive breast milk + formula powder)

6 Mixed feeding (exclusive breast milk + formula powder) + complementary food

14. Did you get advice from professional medical staff on feeding or postpartum recovery within a few days after delivery? (Single-choice question)

1 Yes

2 No

15. Did you get advice from family or friends on feeding or postpartum recovery within a few days after delivery (Single-choice question)

1 Yes

2 No

16. Except for the baby you are feeding, ____at home (Multiple choice question)

0 no other babies

1 there is a baby less than 2 years old

2 there is also a 2-5 year old baby

3 there is also a 6-10 year old child

4 there is also a child aged 11-16

5 there is a child older than 16 years old

6 currently I am feeding twin (multiple) births

17. Which of the following gave you breastfeeding support? (Multiple choice question)

1 Husband

2 Parents

3 Parents-in-law

4 Hospital experts

5 friends/relatives

6 WeChat discussion groups or other forums related to breastfeeding

7 Network information, WeChat health public account

8 No support for feeding

9 Other ____________

18. Have you been living in Beijing since the first half of this year? (Single-choice question)

1 Yes

2 No

19. Which district of Beijing do you live in? (Fill in the blank question)

________________________

20. What was the status of your family members in the first half of this year? (Single-choice question)

1 Living with husband

2 Living with husband and parents

3 Live with husband and parents-in-law

4 Live alone

5 Other ____________

21. The environment where you live is (Single-choice question)

0 Bungalow

1 Tower apartment (crowded apartment), no garden in the community

2 Tower apartment (crowded apartment), with gardens in the community

3 Apartment building (spacious apartments), no garden in the community

4 Apartment building (spacious apartments), with garden in the community

5 Villa with garden

22. Are there pets in your home (Single-choice question)

1 Yes

2 No

23. How many bedrooms are currently in your home? (Fill in the blank question)

________________________

24. Is there a comfortable space for your baby at home? (Single-choice question)

1 Yes

2 No

25. Are there squares or parks within walking distance? (Single-choice question)

1 There is a square

2 There are parks

3 Both squares and parks

4 There are no squares and parks, only activities in the community

26. During the lockdown period, do you think the space for activities is too small/crowded? (Single-choice question)

1 Yes

2 No

27. The current annual income of your family is approximately (Single-choice question)

1 Less than RMB 200,000 per year

2 200,000-300,000 RMB per year

3 300,000 to 400,000 RMB per year

4 400,000-500,000 RMB per year

5,500,000 or more per year

6 Prefer not to say

7 Other ____________

28. What is your highest degree of education? (Single-choice question)

1 Part-time undergraduate, junior college or below

2 Full-time undergraduate

3 Master's degree students

4 PhD students

29. How many years have you spent in education since the first grade of elementary school? (Fill in the blank question)

________________________

30. What is your current job? (Fill in the blank question)

________________________

31. Which of the following is your dietary preference? (Single-choice question)

1 I like to eat everything

2 Vegetarian (accepts eggs and milk)

3 Semi-vegetarian (no meat, only accepts fish)

4 Vegan (eggs and milk are not accepted)

5 Other ____________

32. Have you had the following symptoms recently: persistent dry cough or high fever (Single-choice question)

1 Yes

2 No

33. Has any other member of your family had a persistent dry cough or high fever recently? (Single-choice question)

1 Yes

2 No

34. Have you received a PCR test for the new coronavirus? (Single-choice question)

1 No

2 Yes, the result was negative

3 Yes, the result was positive

For questions 34-45 below, please select the impact of the pandemic on you

35. What was the impact of the pandemic on your planned feeding method? (Single-choice question)

1 Great impact

2 Moderate impact

3 little impact

4 No impact

36. Did the pandemic impact on your original planned delivery location? (Single-choice question)

1 Yes

2 No

37. The impact on your family income (Single-choice question)

1 Great impact

2 Moderate impact

3 little impact

4 No impact

38. The impact on your purchase of daily necessities (Single-choice question)

1 Great impact

2 Moderate impact

3 little impact

4 No impact

39. Impact on the convenience of purchasing food for your family (Single-choice question)

1 Great impact

2 Moderate impact

3 little impact

4 No impact

40. Impact on the ability of your family to buy medicines (Single-choice question)

1 Great impact

2 Moderate impact

3 little impact

4 No impact

41. What is the impact on your online shopping frequency? (Single-choice question)

1 Significantly increased

2 Increased

3 Basically unchanged

4 Reduced

42. The main categories you purchased online in the first half of this year were

1 Food

2 Clothing or accessories

3 Health Products

4 Daily necessities

5 Medicines

6 Other ____________

43. Did the pandemic affect your work? (Single-choice question)

1 Yes

2 No, I am on maternity leave

3 No, I don’t have a job

4 No, other reasons

44. Did the pandemic affect your spouse's work? (Single-choice question)

1 Yes

2 No, he works at home

3 No, he is unemployed

4 No, other reasons

45. Did you get enough feeding and health support during your stay at home in the first half of the year? (Single-choice question)

1 Enough

2 Not enough

46. In the last week, how often did you engage in the following activities?

|  | 1 time /day | 4-5 times/ week | 1-3 times /week | No such activity |
| --- | --- | --- | --- | --- |
| Shopping |  |  |  |  |
| Go for a walk |  |  |  |  |
| Business trip work |  |  |  |  |
| Participate in online activities |  |  |  |  |
| Consult medical staff about health issues |  |  |  |  |
| Participate in discussions in WeChat groups |  |  |  |  |
| Relax with relaxation equipment (including yoga) |  |  |  |  |

47. During the first half of this year, which of the following descriptions fit your situation

|  | 1 time /day | 4-5 times/ week | 1-3 times /week | No such activity |
| --- | --- | --- | --- | --- |
| Free to chat with friends and family |  |  |  |  |
| Loss of appetite |  |  |  |  |
| Increased appetite Increased diet |  |  |  |  |
| Feel close to the community |  |  |  |  |
| Very tired |  |  |  |  |
| Enjoyed the coming and going of spring |  |  |  |  |
| More time to pay attention to your own health |  |  |  |  |
| Worried every day |  |  |  |  |
| Have time to exercise |  |  |  |  |
| Coping well with the epidemic |  |  |  |  |
| Feeling depressed |  |  |  |  |
| Poor sleep or difficulty falling asleep |  |  |  |  |
| Feeling lonely |  |  |  |  |
| I can't relax well |  |  |  |  |
| Feeling that housework chores are fairy assigned |  |  |  |  |
| Feeling easily irritated |  |  |  |  |
| I feel that I can have more time to spend on my hobbies |  |  |  |  |

48. During the COVID-19 pandemic, how much did you worry about the following options?

|  | Didn’t worry | Worried a bit | Worried a lot | Extremely worried |
| --- | --- | --- | --- | --- |
| My own health |  |  |  |  |
| Health of family members |  |  |  |  |
| Health of other vulnerable groups in society |  |  |  |  |
| My family pressure during this period |  |  |  |  |
