# Supplementary material for: Maternal mental health and well-being during the COVID-19 pandemic in Beijing, China
Source: World J Pediatr. 2021 Jun 25;17(3):280–9. doi: 10.1007/s12519-021-00439-8 (PMC8231088; doi:10.1007/s12519-021-00439-8)
Supplement: Supplementary file 3 — Supplementary Table 1 (DOCX 20 KB) [file 12519_2021_439_MOESM3_ESM.docx]

**Supplementary Table 1** Principal component analysis: rotated component matrix^a^

| Variables | Component | | | |
| --- | --- | --- | --- | --- |
|  | 1 | 2 | 3 | 4 |
| Maternal mental health |  |  |  |  |
| Down mood | **0.846** | -0.009 | 0.078 | 0.056 |
| Lonely | **0.852** | -0.031 | 0.115 | 0.057 |
| Not relaxed | **0.819** | 0.077 | -0.050 | 0.111 |
| Easily annoyed | **0.740** | 0.035 | 0.140 | 0.047 |
| Worry | **0.719** | 0.006 | 0.110 | 0.162 |
| Tired | **0.717** | 0.028 | -0.001 | 0.291 |
| Decreased appetite | **0.590** | -0.086 | 0.152 | 0.445 |
| Bad sleep | **0.829** | 0.064 | 0.018 | -0.002 |
| Coping |  |  |  |  |
| Focus on own health | 0.045 | **0.627** | 0.442 | 0.048 |
| Chatting | -0.082 | **0.702** | -0.067 | 0.202 |
| Enjoy | 0.064 | **0.749** | 0.089 | 0.154 |
| Coping well | 0.048 | **0.744** | 0.249 | -0.182 |
| Time to focus on interests |  |  |  |  |
| Exercise | 0.146 | 0.242 | **0.755** | -0.011 |
| More hobbits | 0.071 | 0.083 | **0.798** | 0.188 |
| Positive reaction to surroundings | | | | |
| Close to community | 0.067 | 0.392 | 0.340 | **0.571** |
| Increased appetite | 0.413 | 0.116 | 0.024 | **0.662** |

Extraction method: principal component analysis (PCA). Rotation method: varimax with Kaiser normalization. ^a^Rotation converged in 5 iterations.

The PCA was conducted on the correlation matrix of the survey responses. Item “Feeling that housework chores are fairy assigned” in the original questionnaire (question 47) was not included into the PCA, instead, it was analyzed as a covariance in the regression.
